# Respiratory virus infection up-regulates TRPV1, TRPA1 and ASICS3 receptors on airway cells

**DOI:** 10.1371/journal.pone.0171681

**Published:** 2017-02-10

**Authors:** Shadia Omar, Rebecca Clarke, Haniah Abdullah, Clare Brady, John Corry, Hanagh Winter, Olivier Touzelet, Ultan F. Power, Fionnuala Lundy, Lorcan P. A. McGarvey, S. Louise Cosby

**Affiliations:** Queen’s University Belfast, Centre for Experimental Medicine, School of Medicine, Dentistry and Biomedical Sciences, Medical Biology Centre, Belfast, United Kingdom; University of Georgia, UNITED STATES

## Abstract

Receptors implicated in cough hypersensitivity are transient receptor potential vanilloid 1 (TRPV1), transient receptor potential cation channel, Subfamily A, Member 1 (TRPA1) and acid sensing ion channel receptor 3 (ASIC3). Respiratory viruses, such *as* respiratory syncytial virus (RSV) and measles virus (MV) may interact directly and/or indirectly with these receptors on sensory nerves and epithelial cells in the airways. We used in vitro models of sensory neurones (SHSY5Y or differentiated IMR-32 cells) and human bronchial epithelium (BEAS-2B cells) as well as primary human bronchial epithelial cells (PBEC) to study the effect of MV and RSV infection on receptor expression.

Receptor mRNA and protein levels were examined by qPCR and flow cytometry, respectively, following infection or treatment with UV inactivated virus, virus-induced soluble factors or pelleted virus. Concentrations of a range of cytokines in resultant BEAS-2B and PBEC supernatants were determined by ELISA.

Up-regulation of TRPV1, TRPA1 and ASICS3 expression occurred by 12 hours post-infection in each cell type. This was independent of replicating virus, within the same cell, as virus-induced soluble factors alone were sufficient to increase channel expression. IL-8 and IL-6 increased in infected cell supernatants. Antibodies against these factors inhibited TRP receptor up-regulation. Capsazepine treatment inhibited virus induced up-regulation of TRPV1 indicating that these receptors are targets for treating virus-induced cough.

## Introduction

Cough involves a complex reflex arc that begins with the stimulation of an irritant receptor and serves as a physiological mechanism protecting against aspiration of harmful substances into the respiratory tract and helping clear the airway of mucus and excessive secretions. However, in disease states such as asthma and chronic obstructive pulmonary disease (COPD) cough can become debilitating [1, 2].

Several types of receptors have been implicated in the cough reflex sensitisation, including the ‘transient receptor potential vanilloid 1 (TRPV1) receptor, transient receptor cation channel, Subfamily A, Member 1 (TRPA1) and the acid sensing ion channel receptor 3 (ASIC3).These receptors are expressed on afferent sensory airway nerves and airway epithelial cells and are activated by inhaled irritants known to induce cough. TRPV1 is sensitized and activated by multiple mediators and intracellular second messengers, including capsaicin, anandamide, lipoxygenase products, serine/threonine protein kinases, and tyrosine phosphorylation [3, 4, 5]. Many inflammatory mediators such as ATP, bradykinin, NGF or PGE_2_ indirectly activate TRPV1 and TRPA1 channels, while activation of these receptors has been shown to induce Il-6 and Il-8 [6, 7]. TRPV1 induces increases in IL-6/IL-8 release through TAK1 activation of JNK1-dependent and JNK1-independent signaling pathways. Their joint activation is required for NF-κB to elicit sufficient positive feedback control of JNK1/2 phosphorylation to induce increases in IL-6/8 release [8]. Therefore these cytokines play a prominent role in TRPV1 and TRPA1 regulation.

ASICs are members of the DEG/ENaC family of sodium ion channels and are activated by a drop in pH [9]. Studies aimed at clarifying the physiological importance of ASICs in pain, neurological and psychiatric disease have been reported [10] but their role in pulmonary hypersensitivity requires investigation. ASIC1a, ASIC2a, and ASIC2b subunits are mainly expressed in the CNS and olfactory bulbs while ASIC1b and ASIC3 subunits predominate in the peripheral nervous system (PNS) including the airway. ASIC3 can produce a sustained current in response to decrease in pH [11].

TRPV1, TRPA1 and ASICS3 receptors are all upregulated by hypoxia [12, 13, 14]. The transcription factor hypoxia-inducible factor 1α (HIF-1α) has been shown to bind to a specific hypoxia response element–like motif in the TRPA1 gene [14] and enhance calcium-gating capabilities under hypoxic conditions [15]. Furthermore, the activity of TRPA1 is regulated by TRPV1, with the converse likely since there is extensive crosstalk between these two channels via calcium and presence of both channels in one cell limits constitutive activity and calcium leakage [16, 17].

Acute cough in healthy adults is almost exclusively associated with respiratory tract infection [18]. In asthmatic patients respiratory viruses are responsible for 85% of asthma exacerbations in children and 75% in adults [19]. In COPD, virus infection is associated with 78% of exacerbations and the outcome is severe resulting in greater drops in lung function, increase in cough and prolonged hospitalisation compared to non-infective exacerbations [18, 20]. Cough can also be a method of spreading the infection and may contribute to the pathology of the disease. We have recently shown that rhinovirus (a positive strand RNA virus in the family *Picornaviridae*), infection causes up-regulation of TRP channels by channel specific-mechanisms. Increase in TRPA1 and TRPV1 levels does not require replication of the virus within the same cell and can be mediated by soluble factors alone [21]. The interaction of other viruses with TRP receptors or viruses with ASICS receptors has not been investigated.

Respiratory syncytial virus (RSV) is one of the major viruses causing the common cold and is the major cause of bronchiolitis in infants [22]. Measles virus (MV) is a respiratory virus which produces a characteristic hacking cough in the majority of patients [23]. Both viruses are negative strand RNA viruses and members of the family *Paramyxoviridae*. In this study we examined whether virus infection by these viruses in bronchial epithelial cells or neuronal cells up-regulates TRPV1, TRPA1 and ASICS3 expression. The cell line BEAS-2B was initially examined and results confirmed in primary human bronchial epithelial cells (PBEC) obtained from healthy subjects. A human epithelial cell derived neuroblastoma cell line SHSY5Y and differentiated IMR-32 cells were used as models of airway nerves. The SHSY5Y cell line consists of neuroblastic cells with multiple, fine, short cell processes [24] and expresses well-characterized neuronal markers, such as neurofilament protein. IMR-32 cells can be differentiated to mature neuronal cells (dIMR-32) and express the neuronal marker MAP-2 as well as TRPV1 and TRPA1 [21, 25]. We also determined whether virus induced receptor up-regulation was mediated directly by replicating virus, within the same cell, or indirectly by virus-induced soluble factors.

## Materials and methods

### Cells and viruses

The human neuroblastoma cells (SHSY5Y) ECACC General Cell Collection, were obtained from Professor Ruth Itzhaki, University of Manchester. The IMR-32 human neuroblastoma cell line was purchased from the European collection of cell cultures (detailed in the supporting information, S1 File). Differentiated IMR-32 cells (dIMR-32) were derived according to the published protocol [25]. Primary human bronchial epithelial cells were obtained by bronchial brushing carried out by medical staff in Belfast City Hospital. Patients who were referred to the respiratory service at the Belfast City Hospital and who were required to undergo flexible bronchoscopy for various reasons were screened for inclusion in the present study. Smokers had normal lung function testing with no history of lung disease and a smoking history of at least 10 years. Subjects who had a respiratory tract infection or exacerbation of their chronic airway disease within the previous 8 weeks, and those receiving systemic corticosteroids during the previous 12 weeks were excluded from the study. All patients gave written informed consent and the study was approved by the Office for Research Ethics Committees Northern Ireland (ORECNI). Following inclusion, patients underwent a bronchoscopy, using guidelines as detailed by the British Thoracic Society [26]. The human bronchial epithelial cell line, BEAS-2B, PBEC, SHS5Y5 and dIMR-32 cells were used for receptor studies. The Edmonston MV vaccine strain was grown and titred in Vero cells. The RSV A2 strain was grown and titred in HEp-2 cells. Detailed methods are given in the supporting information, S1 File.

### Infection and treatment of cells

Cultures of BEAS-2B, PBEC and SHSY5Y cells were initially infected with RSV or MV at selected multiplicities of infection (MOI). MOIs giving the highest level of TRPV1 and ASIC3 mRNA were used in further experiments. Cultures were also either mock infected (with medium alone), treated with, acidified medium, prepared by adding HCl (pH 5.9), capsaicin (10 μM, TRPV1 agonist, Tocris Bioscience, UK), capsazepine (TRPV1 antagonist, Sigma Aldrich Ltd, UK), supernatant from mock cells, virus-free supernatant from infected cells, UV-inactivated standard stock virus or with UV-inactivated pelleted virus (obtained by ultra centrifugation). All of the above preparations were used to determine whether soluble factors present in the supernatant and/or direct interaction of virus particles with the cells are responsible for the modulation of expression of the ion channels. Cell culture medium was buffered and pH monitored to confirm stability. Detailed methods are given in the supporting information, S1 File.

### Immunofluorescence and flow cytometry

Antibodies used for immunofluorescence (IF) and/or flow cytometry were:- Monoclonal anti-measles (IgG1, Oxford Biotechnology, dilution 1:100) and secondary antibodies Alexa Fluor 488 goat anti-mouse (Molecular Probes, dilution 1:500) was used for MV. Palivizumab 100 mg/ml was used as primary antibody for RSV (1:1000 dilution) and polyclonal rabbit anti-human (Dako, dilution 1:200) as secondary antibody. Rabbit polyclonals to ASIC3, TRPV1 and TRPA1 (Abcam. dilution 1:50) or non-immune rabbit serum (as control, dilution 1:50) were used with secondary antibody goat polyclonal to rabbit IgG (Chromeo™ 488, dilution 1/500). Detailed methods for both IF and flow cytometry are given in the supplementary information S1 File.

### RT-PCR and qRT-PCR

TRPV1 and ASICS3 mRNA production expression in mock-infected and infected BEAS-2B, SHSY5Y cells and PBEC was initially examined using RT-PCR. Quantitative RT-PCR was carried out for TRPV1, ASICS3, TLR2, TLR3, and TLR4, using a QuantiTect Reverse Transcription Kit (Qiagen) for cDNA production. Details of primers and reaction conditions are given in the supporting information S1 File.

### Treatment of cell cultures with anti-receptor antibodies

BEAS-2B and SHSY5Y cell cultures in 24 well plates were treated with anti-TRPV1 or anti-ASIC3 (rabbit polyclonal, dilution 1:100) or with non-immune rabbit serum for 2 hours prior to infection or mock infection. Anti-receptor antibodies were left in the cultures throughout the duration of the experiment.

### Enzyme-Linked Immunosorbent Assay (ELISA)

The Human Inflammatory Cytokines & Chemokines Multi-Analyte ELISArray Kit MEH-004A (SABiosciences) was used. The cytokines and chemokines represented in the array were IL-1α, IL-1β, IL-2, IL-4, IL-6, IL-8, IL-10, IL-12, IL-17A, IFNg, TNFa, and GM-CSF.

### Treatment of cell supernatants with anti-IL6 and anti-IL8 antibodies

Virus induced supernatants from infected cells were treated with rabbit anti-IL-6 or rabbit anti-IL8 or (normal rabbit serum) control antibodies (all from Abcam) at concentrations specified for 1 hr before treatment of cell monolayers. Treated supernatents were left in place throughout the experiment.

### Statistical analysis

All values are presented as the mean of at least 3 independent experiments unless otherwise stated. Statistical analysis was performed by using the one-way analysis of variance (ANOVA) followed by Tukey’s post-hoc test for multiple comparisons of groups. Values of p<0.05 were considered statistically significant and indicated by *p<0.05, **p<0.01; ***p<0.001.

## Results

### TRPV1 and ASICS 3 expression and infection in BEAS-2 and SHSY5Y cells

**Fig 1:**
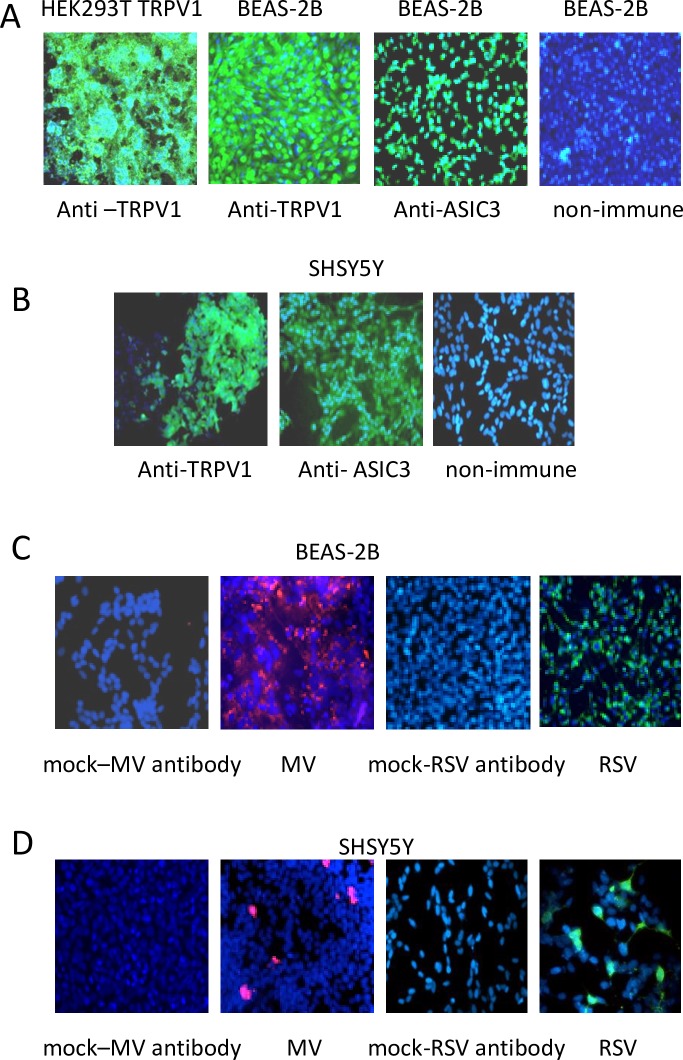
BEAS-2B and SHY5Y5 cells express TRPV1 and ASIC3 and support virus replication. (A) BEAS-2B and (B) SHSY5Y cells were stained with anti-TRPV1, anti-ASIC3 antibodies or non-immune rabbit serum and DAPI. HEK293T TRPV1 transfected cells were used as positive control for TRPV1 expression. Receptors (green), nuclei (blue). (C) BEAS-2B cells were mock infected or infected with RSV or MV at an MOI of 1 for 48 (D) SHSY5Y cells were mock infected or infected with RSV or MV at an MOI of 1 for 72 hours and stained with anti-viral antibodies and DAPI. RSV (green), MV (red), nuclei (blue). Magnification X 200.

To determine whether TRPV1 and ASIC3 are expressed in BEAS-2B and SHSY5Y cells coverslips cultures were stained with anti-TRPV1, anti-ASIC3 antibodies or non-immune rabbit serum as control. HEK293T TRPV1-transfected cells were used as positive controls for TRPV1 expression. Fig 1A and 1B show expression of both receptors in both cell types. TRPV1 and ASIC3 RT-PCR products were obtained from each cell line and confirmed by DNA sequencing (not shown). BEAS-2B cells and SHSY5Y cells were infected at an MOI of 1 for 24 hours with MV or RSV and stained with anti-viral antibodies confirming infection (Fig 1C and 1D). No staining was seen in mock infected cultures (not shown).

### RSV- and MV-induced TRPV1 and ASICS3 channel mRNA up-regulation in BEAS-2B and SHSY5Y cells is dependent on virus multiplicity of infection and time post-infection

**Fig 2:**
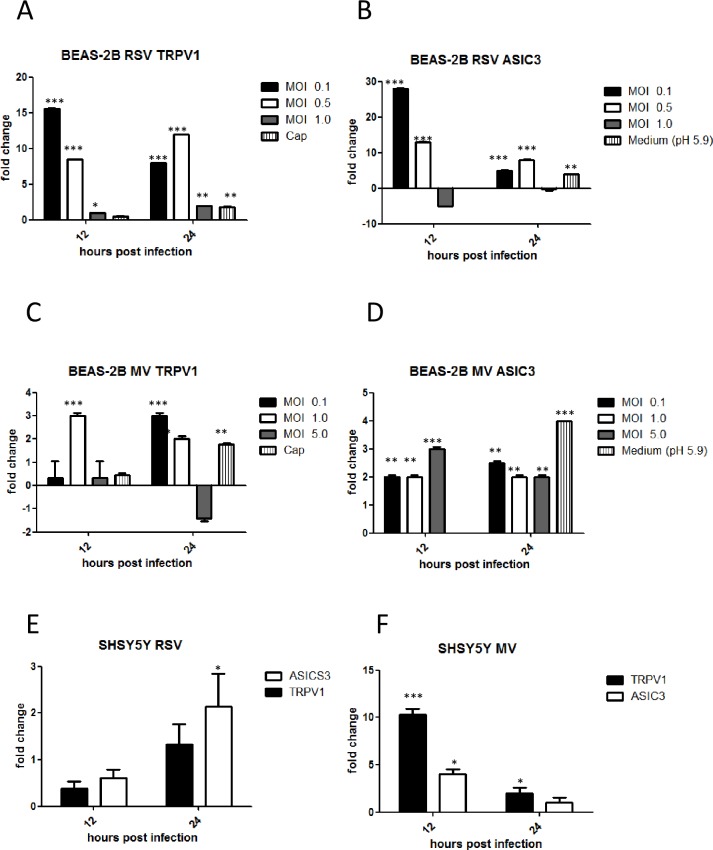
RSV and MV induced TRPV1 and ASIC3 mRNA up-regulation is dependent on virus titre and time post infection. BEAS-2B cells were infected at MOIs of 0.1, 0.5 and 1 (RSV) and 0.1, 1 and 5 (MV) for 12, 24 and 36 hours. Capsaicin (10μM) was used as positive control for stimulation of TRPV1 and acidified medium (pH 5.9) for stimulation of ASICS3. SHSY5Y were also infected with RSV (MOI 0.1) and MV (MOI 1) for 12 and 24 hours. TRPV1 and ASIC3 mRNA levels were determined by qRT-PCR. (A), (B) (C) and (D) BEAS-2 B cells; (E) and (F) SHSY5Y cells. (A) RSV TRPV1, (B) RSV ASCI3, (C) MV TRPV1, (D) MV ASICS3, (E) RSV and (F) MV. Data are presented as relative fold changes to TRPV1 and ASIC3 mRNA levels in uninfected/untreated control cultures.

We performed preliminary experiments to determine if MV and RSV infection induced TRPV1 and ASIC3 channel mRNA up-regulation in BEAS-2B and SHY5Y5 cells was related to the inoculum titre and/or duration of infection. Cells were infected at MOIs of 0.1, 0.5 and 1 (RSV) and 0.1, 1 and 5 (MV) for 12, 24 and 36 hours. Capsaicin, a TRPV1 agonist was used as positive control for stimulation of TRPV1 and acidified medium for stimulation of ASICS3. It was only possible to maintain a pH of 5.9 for 24 hours as further pH reduction was toxic for cells. RSV induced a 15-fold increase in TRPV1 and almost 30-fold increase in ASICS3 mRNA in BEAS-2B cells, respectively, at 12 hours post infection (hpi) (Fig 2A and 2B). Levels of receptor mRNA also increased after MV infection but to a lesser degree, 3-fold for both TRPV1 and ASIC3 at 24 hpi (Fig 2C and 2D). Maximal expression for both TRPV1 and ASIC3 channels at 12 hpi was noted at a MOI of 0.1 for RSV. For MV the highest expression of TRPV1 occurred at a MOI of 1.0 and ASICS3 at a MOI of 5. A MOI of 0.1 for RSV and MOI of 1 for MV were chosen for all further studies. At 36 hpi (not shown) or in some cases at the highest MOIs tested for each virus, infection resulted in cell death with concomitant drops in TRPV1 and ASICS mRNA levels.

SHSY5Y neuronal cells were infected by RSV or MV at a MOI of 0.1 and 1, respectively, and receptor mRNA fold changes determined as before. Following RSV infection only a 2-fold change for TRPV1 was significant at 24 hpi (Fig 2E). TRPV1 mRNA levels increased by 10-fold and ASIC3 mRNA by 2.5-fold at 12 hpi following MV infection (Fig 2F).

### TRPV1, TRPA1 and ASICS3 protein expression is up-regulated by virus infection

**Fig 3:**
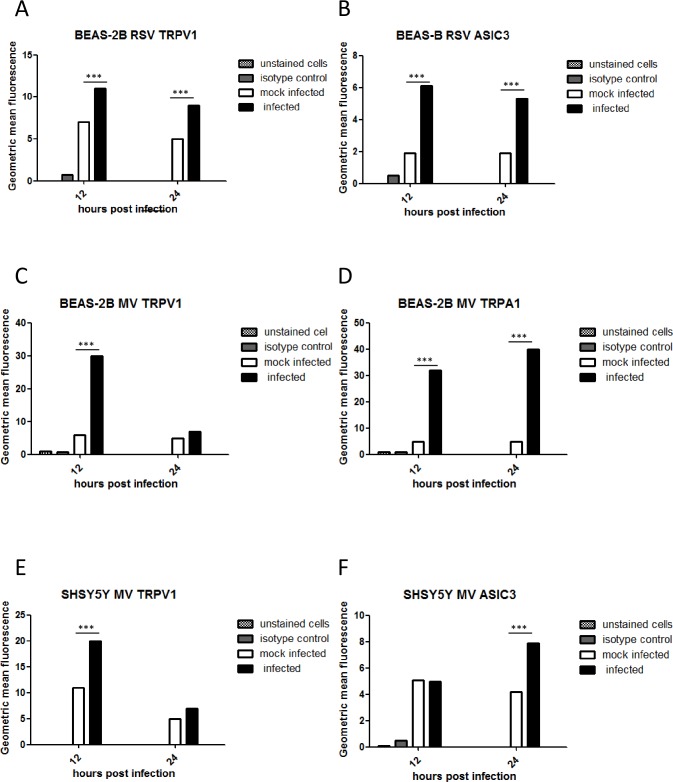
TRPV1, TRPA1 and ASICS3 protein expression is up-regulated by virus infection. (A to D) BEAS-2B cells were infected with RSV (MOI 0.1) and MV (MOI 1). (E and F) SHSY5Y cells were infected with MV (MOI 1). TRPV1, TRPA1 or ASIC3 expression was determined by flow cytometry at 12 and 24 hpi. The levels were quantified by geometric mean fluorescence intensity (GMFI). (A) RSV TRPV1, (B) RSV ASCI3, (C) MV TRPV1, (D) MV TRPA1. The results are representative of 3 independent experiments.

To examine if TRPV1 and ASIC3 protein expression levels also increased after virus infection, BEAS-2B (RSV and MV) and SHSY5Y (MV only) cells were infected for 12 and 24 hours at the chosen MOIs for each virus and flow cytometry carried out. We also examined TRPA1 receptor expression in MV-infected BEAS-2B cells. Increases in protein levels as determined by geometric mean fluorescence intensity (GMFI) were mainly consistent with mRNA levels. TRPV1 and ASICS3 expression in BEAS-2B cells was up-regulated at both 12 and 24 hours after RSV infection (Fig 3A and 3B). TRPV1 was up-regulated 12 hours after MV infection (Fig 3C) but there was no significant change in ASICS3 expression (not shown). TRPA1 levels were similar to those of TRPV1 after MV infection except expression remained high at 24 hpi (Fig 3D). In SHSY5Y cells MV TRPV1 levels increased at 12 hpi (Fig 3E) and ASICS at 24 hpi (Fig 3F). There was no significant up-regulation of either channel by RSV in these cells (not shown).

### UV inactivated virus up-regulates TRPV1 and ASIC3 mRNA and protein expression in BEAS-2B and SHSY5Y cells

**Fig 4:**
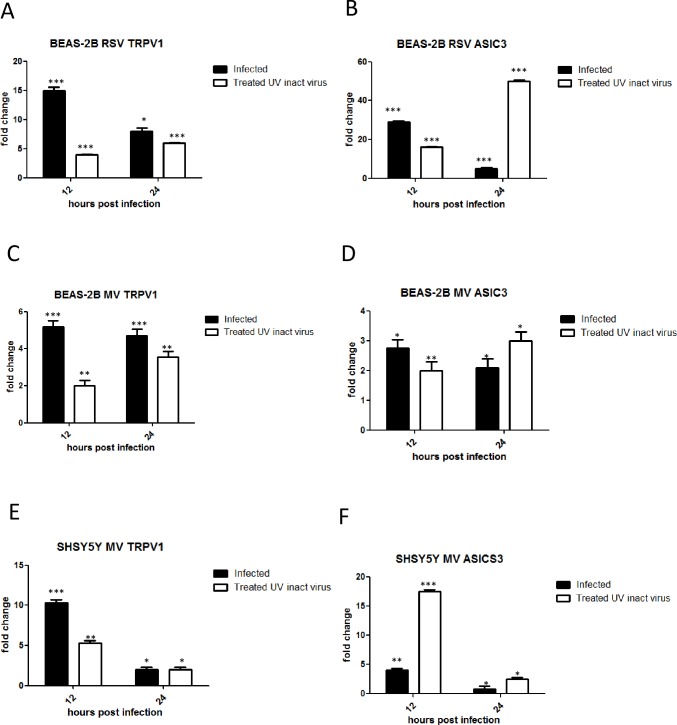
UV inactivated virus preparations up-regulate TRPV1 and ASICS3 mRNA levels. BEAS-2B cells were infected with RSV (MOI 0.1) or MV (MOI 1), or were treated with equivalent virus preparations that had been UV inactivated. SHSY5Y were also infected with MV or treated with UV-inactivated virus. (A, B, C and D) BEAS-2B cells, (E) and (F) SHSY5Y cells. (A) RSV TRPV1, (B) RSV ASIC3, (C) and (E) MV TRPV1, (D) and (F) MV ASIC3. Data is presented as relative fold change to TRPV1 and ASIC3 mRNA levels relative to uninfected/untreated control cultures.

**Fig 5:**
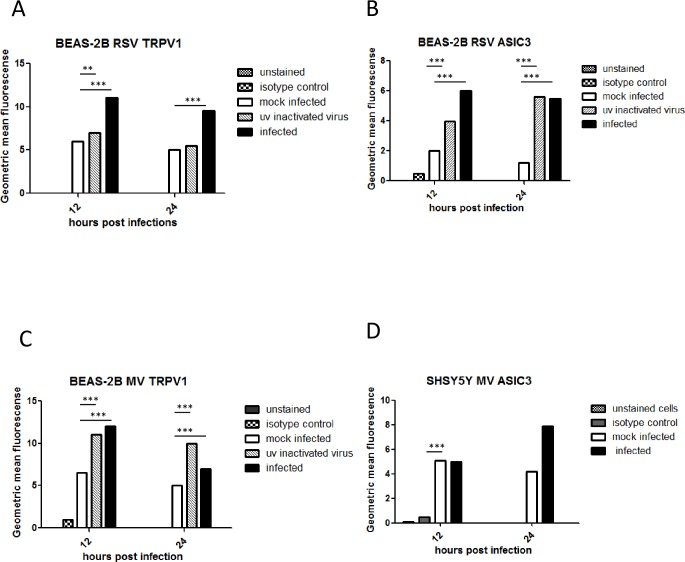
UV Inactivated virus preparations increase TRPV1 and ASICS3 protein expression in BEAS-2B cells. BEAS-2B cells were infected with RSV (MOI 0.1) or MV (MOI 1), or were treated with equivalent virus preparations that had been UV inactivated. TRPV1 and ASIC3 expression was determined by flow cytometry at 12 and 24 hpi. Protein expression levels were quantified by geometric mean fluorescence intensity (GMFI) using Flow cytometry. The results are representative of 3 independent experiments.

We sought to determine if virus replication was necessary to induce up-regulation of TRPV1 and/or ASIC3 channels within the same cell. We therefore compared their mRNA levels after treatment of BEAS-2B (RSV and MV) and SHSY5Y (MV only) cells with infectious and UV-inactivated viruses. TRPV1 and ASICS3 mRNA was up-regulated at both 12 and 24 hours by both infectious and UV-inactivated virus preparations in BEAS-2B cells (Fig 4A–4D). However, TRPV1 fold changes were lower in BEAS-2B cells exposed to UV-inactivated RSV or MV at 12 hpi. UV inactivated MV was also found to up-regulate TRPV1 and ASICS3 mRNA levels in SHSY5Y cells (Fig 4E and 4F). Flow cytometry of BEAS-2B cells infected or treated with UV-inactivated viruses demonstrated that protein expression levels were, for the most part, consistent with mRNA fold changes (Fig 5A–5D).

### TRPV1 and ASIC3 mRNA levels are up-regulated by virus induced soluble factors and UV inactivated pelleted virus

**Fig 6:**
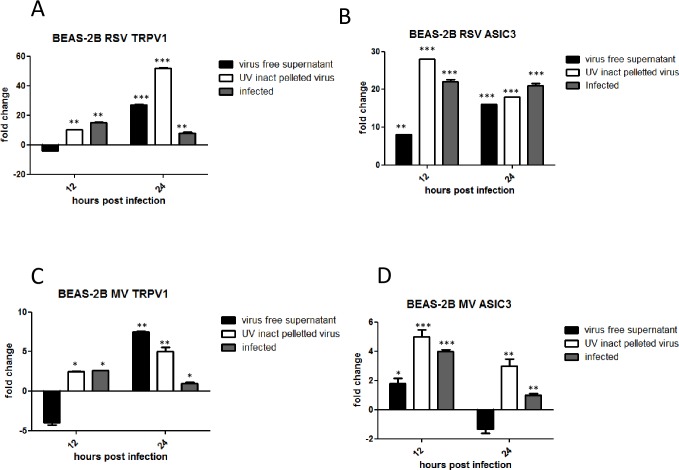
TRPV1 and ASIC3 mRNA levels are up-regulated by virus-induced soluble factors and UV inactivated pelleted virus. BEAS-2B cells were infected with RSV (MOI 0.1) and MV (MOI 1) or treated with virus free supernatant, supernatant from mock infected BEAS-2B cells or UV inactivated pelleted virus. TRPV1 and ASIC mRNA levels were quantified by qRT-PCR. (A) RSV; TRPV1, (B) RSV; ASICS3 (C), MV; TRPV1 (D) MV; ASIC3. Data is presented as relative fold change to TRPV1 and ASIC3 mRNA levels in uninfected/untreated control cultures.

As our findings suggested that TRPV1 and ASIC3 up-regulation was not solely dependent on replicating virus, we determined whether this up-regulation was due to binding/entry of non-replicating virus particles in cells and/or the presence of virus-induced soluble factors in the inoculum. BEAS-2B cells were treated with ultracentrifuged virus-free supernatant or UV inactivated pelleted virus, prepared from MV or RSV-infected BEAS-2B cells (as described in the supplementary information) and diluted to equivalent levels as infectious RSV (MOI 0.1) and MV (MOI 1). Cultures were also infected with live virus for comparison. TRPV1 mRNA levels were up-regulated by 28-fold and 7-fold at 24 hpi by virus-free supernatant from RSV and MV infected cells, respectively. ASIC3 mRNA levels were also up-regulated by virus-free supernatants but to a lesser extent than TRPV1. UV-inactivated pelleted RSV and MV preparations were also able to increase virus up-regulation of both TRPV1 and ASIC3 receptors at 12 and 24 hpi. Again levels were considerably higher for RSV than MV (Fig 6A–6D).

### Treatment of BEAS-2B and SHSY5Y cells with anti-TRPV1 and anti-ASIC3 antibodies inhibits receptor expression up-regulation

**Fig 7:**
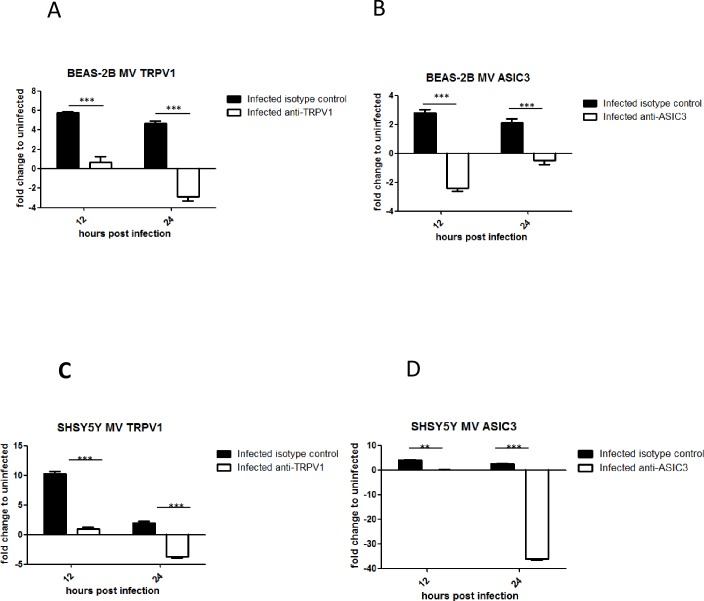
Antibodies to TRPV1 and ASIC3 inhibit up-regulation of these receptors by virus infection. BEAS-2B and SHY5Y5 cells were treated for 1 hour with either anti-TRPV1 or anti-ASIC3 antibodies or with non-immune rabbit serum before infection with MV (MOI 1) or mock infection. Antibodies were left in the culture throughout the experiment. TRPV1 and ASICS3 mRNA levels were determined by qRT-RT. (A) and (B) BEAS-2B cells; (C) and (D) SHSY5Y. (A) and (C) TRPV1, (B) and (D) ASIC3. Fold change was determined relative to uninfected, untreated cells.

The results above indicated that the up-regulation of TRPV1 and ASIC3 expression is independent of virus replication, within the same cell, as virus-induced soluble factors alone or UV inactivated pelleted virus, increased receptor mRNA levels. Therefore, we determined whether the interaction of virus and/or virus induced factors with TRPV1 and ASIC3 receptors can be blocked by anti-receptor antibody treatment. This would be expected to inhibit the up-regulation of receptor mRNA levels as the stimulating factor (either virus components and/or virus induced factors) would be unable to directly interact with the receptor due to antibody blockage. BEAS-2B and SHSY5Y cells were infected with MV or mock infected and treated with anti-TRPV1, anti-ASICS3 or non-immune rabbit serum. Antibody treatment of mock infected cells had no effect on TRPV1 and ASIC3 mRNA levels and did not block virus infection (not shown). However, both anti-receptor antibodies completely inhibited up-regulation of the receptors in both infected BEAS-2B (Fig 7A and 7B) and infected SHSY5Y cells (Fig 7C and 7D) suggesting that these antibodies blocked binding of either virus and/or virus-induced factors to TRPV1 and ASIC3 receptors.

### Toll-like receptors are up-regulated by virus infection in BEAS-2B and SHSY5Y Cells

**Fig 8:**
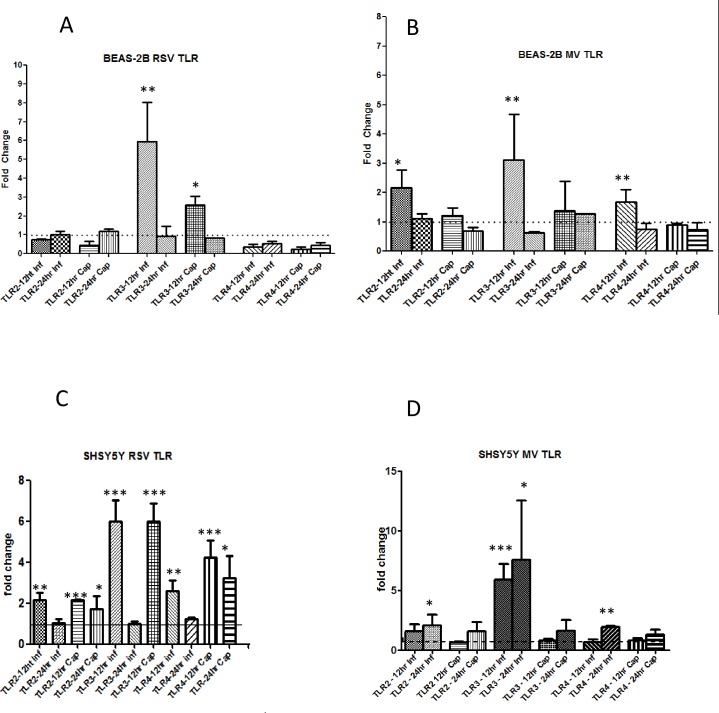
RSV and MV infection induce cell surface TLRs in BEAS-2B and SHSY5Y cells. BEAS-2B and SHSY5Y cells were infected with RSV (MOI 0.1) and MV (MOI 1) or treated with capsaicin (10 μM) for 12 and 24 hours. TLR2, TLR3 and TLR4 levels were determined by qRT-PCR. Data is presented as relative fold change to TLR mRNA levels in uninfected/untreated control cultures. (A) and (B) BEAS-2B cells, (C) and (D) SHSY5Y cells. (A) and (C) RSV, (B) and (D) MV.

Since UV-inactivated pelleted RSV and MV preparations alone were able to increase virus up-regulation of both TRPV1 and ASIC3 receptors it was of interest to determine if cell surface Toll-like receptors (TLRs) were induced by virus infection in BEAS-2B and SHSY5Y cells. This interaction would lead to induction of inflammatory mediators which in turn could up-regulate TRP and ASICS receptors. Cells were infected as before with RSV or MV and mRNA levels of the cell surface receptorsTLR2 and TLR4 determined. TLRL3 was included for comparison as an endogenous TLR responding to double stranded RNA. Cells were also treated with capsaicin to determine if TRPV1 up-regulation had an effect on TLR mRNA levels. Both RSV and MV up-regulated TLR3 mRNA levels 6 and 3-fold, respectively, at 12 hpi in BEAS-2B cells (Fig 8A and 8B). TLR2 was also up-regulated by MV at 12 hpi (Fig 8B). In SHSY5Y cells TLR 2, 3 and 4 were all up-regulated by both viruses (Fig 8C and 8D). Capsaicin treatment resulted in up-regulation of TLR2 and TLR4 in RSV infected SHSY5Y cells.

### TRPV1 and ASIC3 mRNA are up-regulated in virus-infected PBEC cultures

**Fig 9:**
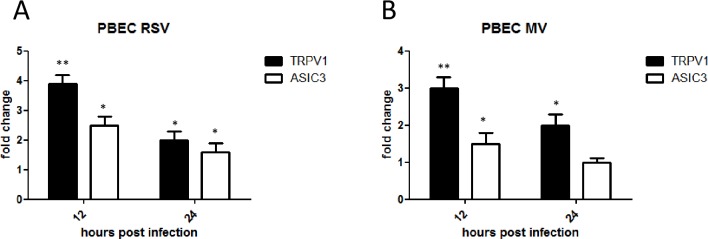
RSV and MV infection induce TRPV1 and ASIC3 mRNA up-regulation in PBEC. PBEC were infected with RSV (MOI 0.1) and MV (MOI 1). TRPV1 and ASIC3 mRNA levels were determined by qRT-PCR at 12 and 24 hpi. (A) RSV), (B) MV. Fold change was determined relative to uninfected/untreated cells.

To determine if the findings in BEAS-2B cells were representative of primary human bronchial epithelial cells we examined TRPV1 and ASICS3 mRNA levels after infection of PBEC with RSV or MV as before. Both receptors were significantly up-regulated in infected PBECs compared to mock-infected controls. RSV infection induced a 4-fold mean increase for TRPV1 and 3-fold for ASIC3 at 12 hpi. TRPV1 mRNA levels increased by 3-fold at 12 hpi but not significantly for ASIC3 following MV infection (Fig 9A and 9B)

### Neutralisation of IL-6 and IL-8 in virus induced supernatant preparations inhibits increase in expression of TRPA1 in dIMR-32 cells

**Fig 10:**
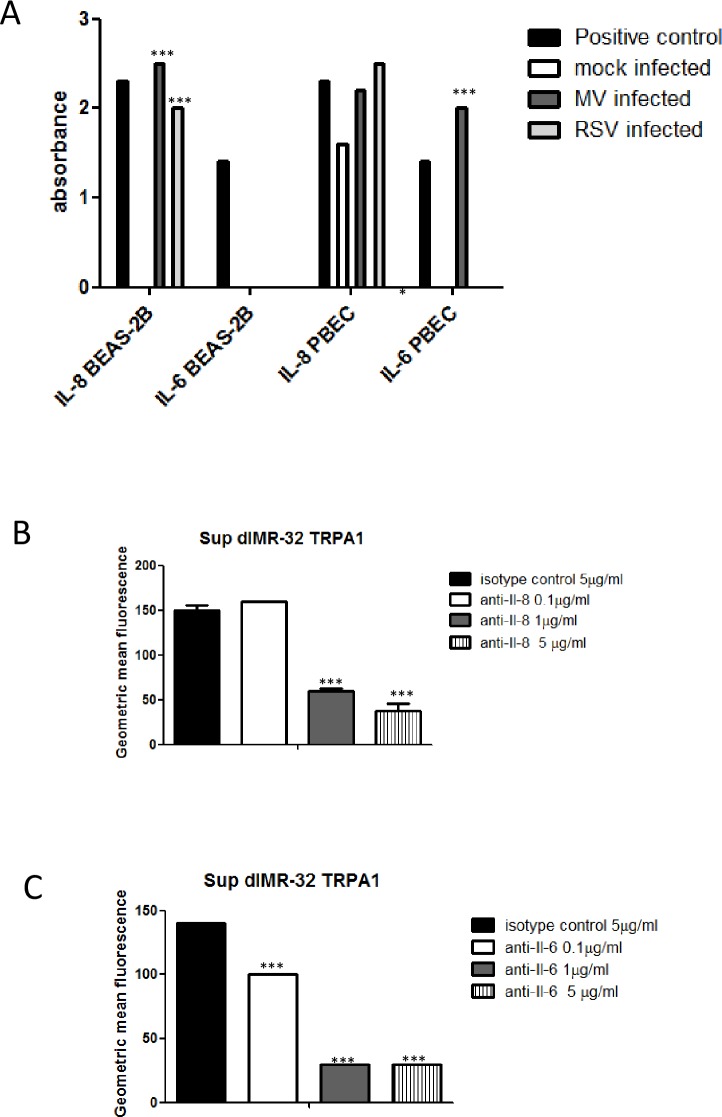
Neutralisation of IL-6 and IL-8 in virus induced supernatant preparations inhibits increase in expression of TRPA1 in dIMR-32 cells. (A) Virus free supernatants from BEAS-2B cells and PBECs infected with RSV (MOI 0.1) and MV (MOI 1) for 24 hours were examined using the Human Inflammatory Cytokines & Chemokines Multi-Analyte ELISArray Kit MEH-004A. Optical density measurements were made for triplicate samples. Significance levels in infected samples were determined in comparison to supernatants from mock infected controls. Virus free supernatants were treated with 0.1, 1 or 5μg /ml of (B) anti- IL-6 or (C) anti-IL-8 or with 5 μg/ml of isotype control antibody. The results are representative of 3 independent experiments.

Virus infection through TLR interaction induces cytokines including Il-6 and Il-8. Therefore the supernatants from RSV and MV infected and mock infected BEAS-2B cells and PBECs were examined for these cytokines and other inflammatory mediators at 24 hpi. In BEAS-2B cells infected with MV, IL-8 was present (2.5 absorbance units) compared to the positive control (2.3 absorbance units) but not detected in mock infected cells. In BEAS-2B cells infected with RSV similar results were observed where IL-8 was present (2 absorbance units). In mock infected PBEC supernatants, IL-8 was present (>1.5 absorbance units), however, this increased to 2.2 and 2.5 absorbance units after MV and RSV infection, respectively. IL-6 was also detected in the supernatant of MV (2.2 absorbance units compared to 1.4 in the positive control) but not RSV infected or mock infected PBEC (Fig 10A). Other inflammatory mediators were not detected for either cell type (not shown).

To determine if IL-6 and/or IL-8 in virus induced supernatants are likely to be responsible for the up-regulation of TRP receptors on airway nerve cells the differentiated dIMR-32 neuronal model was used and levels of TRPA1 (previously shown to be expressed on these cells [25]) were examined. Virus-free supernatants were treated with 0.1, 1 or 5 μg/ml of anti-IL-6 or anti-IL8 or 5μg/ml of isotype control antibody and added to dIMR-32 cell cultures. Flow cytometry for TRPA1 was carried out and the results expressed as GMFI. Both 1 and 5 μg/ml of anti-IL8 and anti-IL6 resulted in similarly large reductions in expression of TRPA1. 0.1 μg/ml of anti-Il-6 also caused a significant reduction, but to a lesser degree than higher concentrations of antibody (Fig 10B and 10C).

### Capazepine treatment of virus infected cells inhibits up-regulation of TRPV1

**Fig 11:**
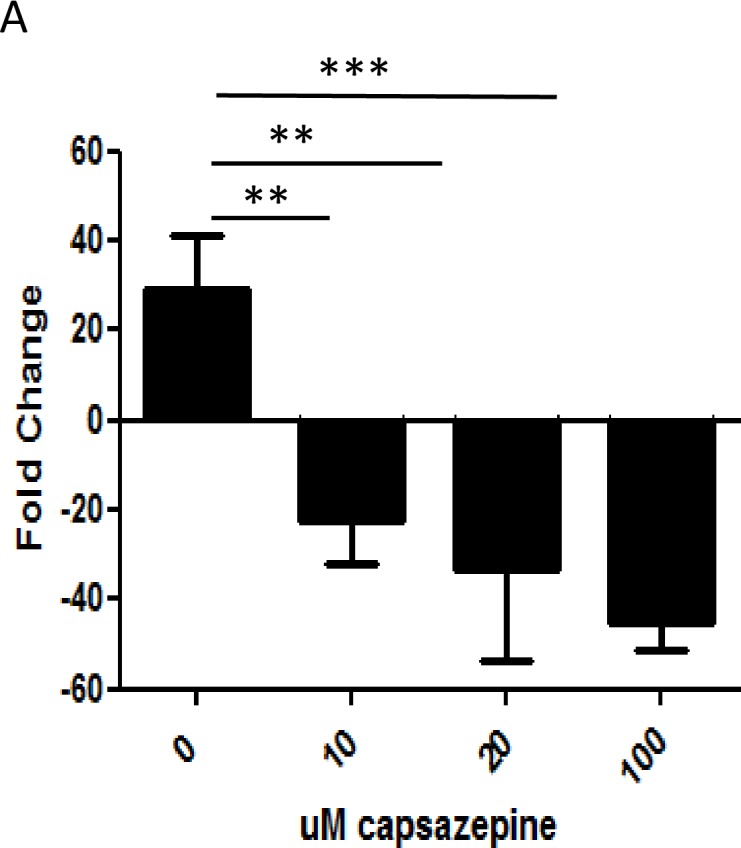
Capazepine treatment of RSV infected BEAS-2B cells inhibits up-regulation of TRPV1. BEAS-2B cells were infected with RSV (MOI 0.1) and treated with 10, 20 or 100 uM capazepine or buffer. TRPV1 RNA levels were determined by qRT-PCR at 12 and 24 hpi.

We wished to determine if the TRPV1 antagonist capazepine would be effective as a treatment during virus infection. BEAS-2B cells were infected with RSV (MOI 0.1) or mock infected and treated with buffer alone or with 10, 20 or 100 μM capazepine for 24 hours. TRPV1 mRNA levels were significantly reduced in a dose-dependent manner to levels below those in mock infected cells (Fig 11).

## Discussion

TRPV1 is strongly implicated in the regulation of irritant-induced airway responses and in particular cough [5, 27]. Expression is increased in the airways of patients with chronic cough [28]. Furthermore, exposure to capsaicin (8-methyl-n-vanillyl-6-nomamide) the chemical extract from hot chilli peppers that evokes cough, also elevates TRPV1 expression [5,28]. Therefore TRPV1 represents a potential candidate receptor in airway hypersensitivity [29]. Up-regulation of ASIC3 was found in inflamed human intestine as inflammation causes tissue acidosis. ASIC3 is the most sensitive nociceptive ion channel responding to acid [30] and is likely to have a role in the cough reflex. Evidence is also emerging that antagonist treatment down-regulates TRPV1 expression as well as inhibiting intracellular [Ca^2+^]_i_ indicating that measurement of the mRNA and/or protein levels are effective in prediction of drug efficacy [31, 32].

We demonstrate here that the infection with MV and RSV in BEAS-2B epithelial and SHSY5Y neuronal cells induces significant up-regulation of TRPV1 and ASIC3 mRNA levels compared to mock-infected cultures. This was confirmed in primary human bronchial epithelial cells demonstrating that BEAS-2B provides a suitable model for further studies of these receptors. All medium in our study was buffered and pH remained stable suggesting that the up-regulation of both TRPV1 and ASIC3 observed are likely due to factors other than acidosis induction.

Our data show that the virus MOI and duration of infection are important factors in TRPV1 and ASIC3 expression modulation in both epithelial and neuronal cells. Both RSV and MV infect fully differentiated airway epithelial cells. However, MV exits but does not enter the lungs by this route but instead through dendritic cells as the relevant epithelial cell entry receptor PVRL4 (nectin 4) is on the basal and not the apical surface of epithelial cells [33, 34, 35]. RSV has been shown to infect pulmonary neurons in mice [36] and it was recently reported that the virus can spread from the airways to the CNS [37]. MV infection of pulmonary nerves has not been investigated to our knowledge but the virus causes several CNS complications with neurons being extensively infected [38]. Therefore, infection of pulmonary nerves in the human airway by both RSV and MV is likely and would lead to direct up-regulation of TRP and ASICS receptors. However, our results indicate that Infection of pulmonary nerves by MV or RSV would not be necessary to provoke the cough reflex as virus replication is not required within the same cell for receptor up-regulation. Instead virus-induced soluble factors. which can be produced from other infected cell types in the airways or UV inactivated pelleted virus, which mimics virus binding and entry without replication, are both effective.

IL-8 was induced by both viruses in BEAS-2B cells and PBECs and MV also induced IL-6 in the latter. It is possible that these cytokines and other factors (not tested in this study) cause TRPV1, TRPA1 and ASIC3 up-regulation. Grace and colleagues observed that TRPV1 antagonists caused partial inhibition of the tussive response to prostaglandin E2 (PGE2) and bradykinin [39]. It has also been recently reported that IL-13 up-regulates TRPV1 in murine bronchial epithelial cells [40]. This is in agreement with a previous study where we showed that soluble factors induced by human rhinovirus infection of fibroblasts up-regulated TRPA1 in the dIMR-32 neuronal cells (21). In the current study we further demonstrate that neutralising both Il-6 and Il-8 in virus-induced supernatants inhibits up-regulation of TRPA1 in the dIMR-32 neuronal model. It is unknown how inflammatory mediators up-regulate TRP receptors. It has been recently shown that HIF-1α upregulation was reduced in IL-6 KO mice in isolated hepatocytes and that stimulation with a single dose of IL-6 induced a nuclear accumulation of HIF-1α [41]. Due to the HIF-1α responsive element in TRPA1 [14], this suggests a possible mechanism for up-regulation of this receptor. Examination of the interaction of a range of factors with TRP and ASICS receptors therefore merits further investigation.

The observation that UV-inactivated pelleted virus preparations also induced TRPV1 and ASIC3 up-regulation points to the mechanism up-stream from induction of inflammatory mediators. Activation of endosomal TLRs requires RNA virus replication while TLRs on the cell surface respond to specific microbial components, including proteins found on the capsid or envelope of viruses. The haemagglutinin protein of MV is known to interact with TLR2 [42] and the fusion protein of RSV with TLR4 [43]. We demonstrate that these TLRs as well as the endogenous receptor TLR3 are up-regulated at the time of TRP-up-regulation by virus infection dependent on cell type. Therefore, induction of cytokines by interaction of virus proteins with cell surface TLRs is a possible mechanism for up-regulation of TRPV1, ASICS3 and other related receptors without the necessity for direct virus infection.

In conclusion, we have shown that two common respiratory viruses can up-regulate expression of ion channels implicated in cough responses. This provides a possible mechanism of how virus infection causes hypersensitisation in the airways and indicates that TRPV1, TRPA1 and ASIC3, or upstream events leading to their activation, are potential therapeutic targets for the treatment of virus-induced cough. Inhibition of these receptors could therefore be beneficial in attenuating virus-associated asthma and COPD exacerbations.

## Supporting information

S1 File (DOCX)
