# Supplementary material for: Respiratory virus infection up-regulates TRPV1, TRPA1 and ASICS3 receptors on airway cells
Source: PLoS One. 2017 Feb 10;12(2):e0171681. doi: 10.1371/journal.pone.0171681 (PMC5302416; doi:10.1371/journal.pone.0171681)
Supplement: S1 File — (DOCX) [file pone.0171681.s001.docx]

**SUPPORTING INFORMATION -S1**

**Materials and Methods**

**Cell Culture**

Vero cells and were grown in Eagle minimum essential medium, with Earle’s salts (EMEM: Sigma, UK). HEp-2 and BEAS-2B cells were grown in Dulbecco’s Modified Eagle’s Medium (DMEM) 1g/L D-Glucose. SHSY5Y and HEK293T/TRPV1 cells were grown in Dulbecco’s Modified Eagle’s Medium (DMEM) + GlutaMax-1 (GIBCO). All cell media was supplemented with 10% (V/V) heat-inactivated foetal calf serum (HI-FBS) with exception of BEAS-2B culture medium, which was supplemented with 20% (V/V) HI-FBS. Medium for HEK293T/TRPV1 cells was also supplemented with Geneticin Sulphate (GIBCO) at a concentration of 0.98 g/ml. Primary bronchial epithelial cells were grown in DMEM supplemented with 5% FBS and primocin. Maintenance medium was prepared similar to growth medium but supplemented with 1% (v/v) HI-FBS. All media were supplemented with 100 units penicillin/streptomycin (Sigma) and buffered with HEPES. For BEAS-2B cells, the flasks were coated with 1% collagen in PBS and incubated at 37ºC for 2 hours prior to culture.

**Preparation of standard stock virus**

Edm MV was routinely grown in Vero cells and RSV in HEp-2 cells. The virus stocks were titered by 50% tissue culture infectious dose end-point (TCID_50_). Incubation was carried out at 37°C in 5% CO_2_ and cytopathic effect (CPE) examined at 6 days post infection.

**Virus UV inactivation**

Virus pools or supernatants were UV inactivated at 12.11 joules for 10 min by utilizing a cross-linker 250 nm (Syngene, UK) in 1 ml volumes, in 35 mm petri dishes.

**Preparation of mock infected, virus free supernatants and virus pellets**

Pools of virus stocks were ultra-centrifuged at 30,000 rpm in an Optima, Beckman Coulter centrifuge (USA) for 2 h at +4°C. The supernatant was collected and the virus pellet gently washed with phosphate buffered saline (PBS) prior to dilution in fresh medium to the original volume centrifuged. Aliquots of each preparation were UV inactivated and stored at -80°C. All supernatant preparations were titred in Vero (MV) or HEp-2 cells (RSV) by TCID_50_ to confirm that they were free of infectious virus. UV inactivated preparations were diluted to levels equivalent to those of infectious virus preparations. Supernatants from mock infected cells were prepared as for virus-free supernatants.

**Immunofluorescence and flow cytometry**

For immunofluorescence (IF) staining, cells were fixed with 4% paraformaldehyde and permeabilised with 0.1% Triton X-100 in PBS (Sigma). Cells were incubated with blocking solution (0.5% bovine serum albumin in PBS) prior to incubation with primary antibodies. Coverslips were washed 3 times in PBS before incubation with secondary antibody. A further 3 washes were undertaken before coverslips were mounted in vectashield mounting medium with DAPI (Vector Laboratories, USA) and examined using a Nikon Eclipse TE2000-U microscope. Staining for flow cytometry was carried out according to the BD Cytofix/Cytoperm Fixation/Permeablization Kit manufacturer’s instructions (BD, UK) using anti-TRPV1, anti-TRPA1 or anti-ASICS3 antibodies and non-immune rabbit serum as a control (as detailed in main paper). The samples were examined in a FACS Canto II (BD) and analyzed by a FlowJo programme (Oregon, USA). TRP and ASICS3 expression levels were quantified by geometric mean fluorescent intensity (GMFI)

**RNA extraction**

For RNA extraction, 1X10^6^ cells were cultured for 24 h prior to infection in 6 well plates (Nunc^TM^). Cells were infected at selected multiplicities of infection (MOIs). Mock-infected cells were treated with supernatant collected from uninfected BEAS-2 or SHSY5Y cells for each time point and experiments were carried out on at least 3 separate occasions unless otherwise stated. RNA was extracted using an RNeasy mini kit (Qiagen, UK) according to the manufacturer’s instructions and quantified in a nanodrop 1000 spectrophotometer (Thermo Scientific, USA). A QuantiTect Reverse Transcription Kit (QIAGEN, UK) was used for cDNA synthesis with integrated genomic DNA removal.

**RT-PCR**

The 2 step RT-PCR was carried out using a Verso™ RT-PCR kit (Thermo scientific) under the following reaction conditions; denaturation at 94°C for 20 sec, primer annealing at 55°C for 30 sec, primer extension at 72°C for 1 min and a final extension step at 72°C for 5 min (cycles: 30). TRPV1 and ASCIS3 primers and β-actin housekeeping gene was obtained from Euroins MWG GmbH:

TRPV1

Forward 5’- GCCTGGAGCTGTTCAAGTTC-3’

Reverse 5’- GATGAGCATGTTGAGCAGGA-3’

ASIC3

Forward 5’- TGGAGGGACATTGAGGAGAC-3’

Reverse 5’- GCTGGCAAGAAACAAAGGTC-3’

β-actin

Forward: 5’ -GGA CGA GCA AGA GAT GG- 3’

Reverse: 5’ –AGG AAG GAA GGC TGG AAG AG- 3’

The PCR products were examined on a 1% agarose gel.

**qRT-PCR**

Quantitative RT-PCR was carried out using a QuantiTect Reverse Transcription Kit (Qiagen) for cDNA production and analyzed using a Mx3005P machine (Agilent Technologies, USA). Taqman primers and probe kits for human TRPV1, TRPA1 and ASICs (Hs00218912_m1 , Hs00175798_m1 and



Hs00245097_m1) and TLRs 2, 3 and 4 (Hs01551078_m1 and Hs00152939-m and Hs00152932_m1) were obtained from Life Technologies (UK) and used according to the manufacturer’s instructions. cDNA, prepared from TRPV1-transfected HEK293T cells were used to create a standard curve. Samples with a high cDNA concentrations were tested for the other genes by conventional RT-PCR and then used to create a relative standard curve to determine the difference in target quantity between a test sample (infected cells) and a calibrator sample (mock-infected cells). Therefore the relative quantities determined could be reliably compared across multiple plates.
